# Desvenlafaxine-a causative agent of extrapyramidal side effects

**DOI:** 10.1192/j.eurpsy.2025.2095

**Published:** 2025-08-26

**Authors:** K. Anurag, H. Mohapatra, L. Dash

**Affiliations:** 1 Psychiatry , Institute of Medical Sciences and sum hospital , baneswar uhB; 2 Psychiatry , Institute of Medical Sciences and sum hospital , neswar abuhB; 3 Psychiatry , Institute of Medical Sciences and sum hospital , aneswar buhB, India

## Abstract

**Introduction:**

Extrapyramidal side effects due to antipsychotics is very common but antidepressants being the causative factor is very less studied. Among antidepressants escitalopram is the most commonly reported. SSRI’s most commonly cause extrapyramidal side effects than other antidepressants. The major theories are changes in chemical, anatomical and physiological perspectives of neurological system. Reported cases shed light that akathisia occurs most commonly followed by dystonia, parkinsonism and tardive dyskinesia states in antidepressant induced extrapyramidal side effects. Desvenlafaxine (o desmethyl venlafaxine) inhibits reuptake of dopamine, serotonin and norepinephrine. EPS occurs due to inhibitory effect of serotonin on dopaminergic pathway in striatum. Females suffer from it more commonly than men. Increasing age in women,CYP2D6 inhibition by concomittently used drugs can increase the risk. SNRI’s less frequently cause EPS than SSRI’s.Although desvenlafaxine is very well tolerated this rare side effect increases noncompliance and chances of suicide. Drug induced parkinsonism also predicts future chances of parkinsonism. Usage of desvenlafaxine sometimes present to the emergency department as dystonia causing panic among care givers of the patients.

**Objectives:**

To determine desvenlafaxine’s role in causing extraPyramidal side effects

**Methods:**

We report here 8 cases of desvenlafaxine induced extrapyramidal side effects. All follow up cases of depression coming for follow up to dept of psychiatry IMS & Hospital who were on desvenlafaxine was analysed.the patients developong extrapyramidal side effects were detected and detailed evaluation and appropriate management was done for those specific cases..all these cases were collected over a period of last 4 years.

**Results:**

In our case series we bring into light rare occurences of extrapyramidal side effects due desvenlafaxine. 5 out of 8 cases were females.most of the symptoms of developed within 5 days of starting the medicine.4 of these cases resulted in secondary parkinsonism, 3 of them resulted in akathisia and one resulted in acute dystonia post adminstration of desvelafaxine. The average dose of desvenlafaxine in all the cases were within 50-100mg. When after extrapyramidal side effects desvenlafaxine was withdrawn replacement with mirtzapine, escitalopram, sertraline or duloxetine was used instead of it resulting in good symptom reduction of primary illness.

**Conclusions:**

Extrapyramidal symptoms with desvenlafaxine is extremely rare. In our case series we highlighted the importance of a keen eye to check for Extrapyramidal side-effects even with the administration of Antidepressants. Future reasearch is needed to find predictors and exact mechanism of action for the same.

**Disclosure of Interest:**

None Declared

